# A Case of Conception without Intromission

**Published:** 1884-07-20

**Authors:** Edward Cass

**Affiliations:** Dresden, Ohio


### A CASE OF CONCEPTION WITHOUT INTROMISSION.

By EDWARD CASS, M.D., OF DRESDEN, OHIO.

In the Reporter for March 1, 1884, is an extract from the Gazz. degli Ospital, October 24, 1883, by Resconi, discussing the subject of conception. The author combats the old opinion that "spermatozoa can make their way into the uterine cavity, if they have already been abandoned only in the vagina. Pallen, Fraer, Ronget and Kolbert have all shown the falsity of such doctrine. Experiments on the dead body are then related, which seemed to put beyond a doubt that semen can enter the uterus only at the time of coition." I have a case to relate that will put beyond a doubt, in practical minds, that conception *has* taken place where it was impossible for the semen to be thrown into the cavity of the uterus. More than a year ago I was called to see a lady in her accouchement, and, on making digital examination, I found about two inches and a half within the vaginal canal a firm, unyielding band or curtain, entirely grown across the canal. On the pubic side of this curtain was a small orifice through which I could not introduce the end of my little finger. Through this small foramen must have drained the catamenia. It was her third confinement. Her testimony was this: Some time after the second child was born, she discovered this obstruction in the vaginal canal, and spoke of it to her husband before he had intercourse with her. She was delighted with the discovery, for she thought the obstruction would be an unfailing measure to prevent child-bearing. Some inflammatory action had taken place after confinement, and closed the canal as I found it. By force the band was ruptured, and the labor completed. It required considerable force to rupture the septum. Here, then, is an instance where it was impossible during sexual congress to inject the seminal fluid into the uterine cavity.

Here is a fact—a truth. Here is an accidentally discovered theorem that demonstrates the possibility that the uterus may be impregnated when the "spermatozoa have been abandoned only in the vagina." The American medical professors, in their docility to European speculative teachings, are peculiarly credulous. Since I have made this narrated discovery, I have doubt that the seminal fluid is almost never thrown into the uterine cavity. This unyielding curtain had no power to dilate the small orifice that speculators claim for the mouth of the uterus. The corollary is plain, without a doubt, that spermatozoa were, in this case, abandoned quite a distance from the mouth of the uterus. It may be that Pallen, Fraer and Kolbert, *et al.*, would take this case to be an exceptional one to the rule, that the spermatozoa in the Americans are possessed of greater energy to surmount difficulties than those of other countries—and take into consideration that this occurred in *Ohio*, *too*, where we find the most determined, enterprising and aggressive of the race. If this could be copied into every European journal, it might be a cause for them to reflect and revolutionize a little speculative theory.—*Med. and Surg. Rep.*

